# Autochthonous Cases of Tick-Borne Encephalitis, Belgium, 2020

**DOI:** 10.3201/eid2708.211175

**Published:** 2021-08

**Authors:** Anke Stoefs, Leo Heyndrickx, Jonathan De Winter, Evelien Coeckelbergh, Barbara Willekens, Alicia Alonso-Jiménez, Anne-Marie Tuttino, Yvette Geerts, Kevin K. Ariën, Marjan Van Esbroeck

**Affiliations:** Institute of Tropical Medicine, Antwerp, Belgium (A. Stoefs, L. Heyndrickx, K.K. Ariën, M. Van Esbroeck);; University Hospital of Antwerp, Antwerp (J. De Winter, E. Coeckelbergh, B. Willekens, A. Alonso-Jiménez);; University of Antwerp, Antwerp (B. Willekens, A. Alonso-Jiménez, K.K. Ariën);; Vivasso, Villers-Le-Bouillet, Belgium (A.-M. Tuttino);; AZ Zeno Hospital, Knokke-Heist, Belgium (Y. Geerts)

**Keywords:** central nervous system viral diseases, vector-borne infections, tick-borne encephalitis, arbovirus infections, encephalitis, arboviruses, tickborne infectious, viruses, zoonoses, Belgium

## Abstract

We report 3 confirmed autochthonous tick-borne encephalitis cases in Belgium diagnosed during summer 2020. Clinicians should include this viral infection in the differential diagnosis for patients with etiologically unexplained neurologic manifestations, even for persons without recent travel history.

Tick-borne encephalitis (TBE) is a severe viral zoonosis caused by TBE virus (TBEV) (*1*). To date, confirmed locally acquired human TBEV infections have not been reported in Belgium, although the most common vector, the tick *Ixodes ricinus*, is abundant in Belgium and seroprevalence studies have revealed the presence of TBEV antibodies in dogs, cattle, roe deer, and wild boar (*2*,*3*). We report 3 confirmed autochthonous TBE cases, diagnosed at the National Reference Centre (NRC) for Arboviruses (Antwerp, Belgium) during summer 2020.

## The Study

A 48-year-old woman had muscle pain and an elevated body temperature 2 weeks after a tick bite on her right hip. She tested negative for coronavirus disease (COVID-19), and her general practitioner prescribed antimicrobial drugs. A few days later, the patient was hospitalized with asthenia, tremor, drowsiness, and fever. A neurologist determined signs of peripheral facial palsy with brachial weakness and nuchal rigidity. Cerebrospinal fluid (CSF) showed an elevated leukocyte count (37 cells/µL; reference range 0–5 cells/µL). *Borrelia* serology and PCR results were negative. Magnetic resonance imaging (MRI) showed demyelinating lesions and encephalopathy and electroencephalography showed diffuse slow activity. Serum collected on day 5 after illness onset tested positive for TBEV IgM and IgG by immunofluorescence assay (IFA) performed at the NRC. Several months later, the patient still had weakness of her right arm, loss of cognitive function, inability to concentrate, fatigue, and tremor.

A 59-year-old man was admitted to the neurology department of a hospital in Belgium with paraparesis and meningitis. Influenza-like symptoms, including fever, fatigue, myalgia, and headache, had occurred a few days earlier. CSF showed an elevated leukocyte count (371 cells/µL; reference range 0–5 cells/µL). Positron emission tomography (PET) and MRI showed no signs of underlying malignancies or encephalopathy. Infectious diseases screening did not reveal the etiology. The patient recalled a tick bite after a walk in a forest in his neighborhood 2 weeks before symptom onset. *Borrelia* and TBEV serology were added to the differential diagnosis, and TBEV antibodies were detected by IFA performed on serum collected on day 20 after illness onset. The patient went through a severe motor polyradiculitis and was using a wheelchair at discharge. At his last clinical evaluation, 9 months after hospitalization, the patient’s motor skills had clearly improved.

A 58-year-old man sought medical attention 48 hours after onset of dyspnea, cough, and fever. A COVID-19 test was done and repeated a week later; results of both were negative. The symptoms subsided for a week, but then fever returned, accompanied by severe and persistent headaches, weakness, decreased appetite, and diarrhea. The patient lived in the woods and enjoyed outdoor activities, such as biking and hiking. He recalled multiple tick bites and a bite by a sick squirrel in the weeks before symptom onset. A transesophageal echocardiogram and PET scan were normal, and screening for expected infectious diseases was negative. TBE was diagnosed by IFA performed on serum collected on day 18 after illness onset. Except for occasional headaches, he recovered without residual symptoms.

The NRC used Flavivirus Profile 2 (EUROIMMUN AG, https://www.euroimmun.com) mosaic IFA to detect TBEV IgM and IgG antibodies in serum from the 3 patients and in CSF from 2 of them (no CSF was available for case 3). TBEV-specific antibodies were confirmed in all patients by plaque-reduction neutralization test (PRNT) with a 90% PRNT at titer ≥1:25. Retrospective real-time reverse transcription PCR (rRT-PCR), adapted from M. Schwaiger (*4*), on acute-phase serum collected from case-patient 3 revealed the presence of TBEV RNA, but the viral load was too low for further analysis (Table).

**Table:** Laboratory results confirming TBEV infections in 3 autochthonous human cases, Belgium, 2020

Case no.	Symptom onset date	Exposure		Sample type, days after symptom onset	Flavivirus IFA		PRNT_90_ titer	rRT-PCR
		Likely site, postal code	Likely route, time		IgM†	IgG‡		
1	Jun 5	Oostkamp, 8020	Tick bite, 2 wk before symptom onset	Serum, 5	TBEV+	TBEV+	1:25	ND
				CSF, 6	TBEV+	TBEV+	ND	ND
2	Jun 21	Lille, 2275	Tick bite, 2 wk before symptom onset	CSF, 18	TBEV+	TBEV+	ND	ND
				Serum, 20	TBEV+	TBEV+	1:60	ND
3	Jul 20	Wanze, 4520	Multiple tick bites in the weeks before symptom onset	Serum, 2	–	–	ND	+
				Serum, 18	TBEV+	TBEV+	1:194	ND

*CSF, cerebrospinal fluid; IFA, immunofluorescence assay; ND, not done; PRNT_90_, plaque reduction neutralization testing at 90% sensitivity; rRT-PCR, real-time reverse transcription PCR; TBEV, tick-borne encephalitis virus; +, positive; –, negative. †Only TBEV-positive on the flavivirus mosaic IFA. ‡Also positive signal for >1 other flavivirus on the mosaic IFA, including West Nile virus, Japanese encephalitis virus, yellow fever virus, and dengue virus serotypes 1–4.

## Conclusions

We describe 3 cases of confirmed autochthonous TBE in Belgium. TBEV IgM and IgG were detected in serum samples from all 3 cases. TBE was confirmed by PRNT. Intrathecally produced TBEV IgM were detected in 2 cases. In the third case, for which no CSF was available, TBE infection was confirmed by detection of TBEV RNA in an acute-phase serum sample. Because the virus typically is not detectable in serum or CSF by the time patients undergo TBE testing, rRT-PCR was not performed on convalescent samples from the other 2 cases (*4*,*5*). PCR testing on urine can be useful 1–2 weeks after symptom onset (*6*), but urine samples were not available from these cases. 

The 3 cases we describe met the current European Centre for Disease Prevention and Control case definition for confirmed TBE (*7*). None of the patients had been vaccinated against TBEV or other flaviviruses, and none had traveled abroad in the months before symptom onset. Belgium closed its borders during March 20 through mid-June 2020 as part of measures to contain the COVID-19 pandemic. These regulations greatly increased outdoor activities, such as walking in forests, among the population in early spring 2020, probably leading to higher exposure to ticks (*8*). The increased incidence of tick bites also was illustrated through the online platform TekenNet (*9*), a project of the Belgian Institute of Public Health that monitors tick exposure among the population by inviting citizens to voluntarily report tick bites. The 3 patients had been exposed in geographically separate regions of the country, 2 of which were adjacent to an area with known TBEV seropositivity in animals (Figure).

**Figure:**
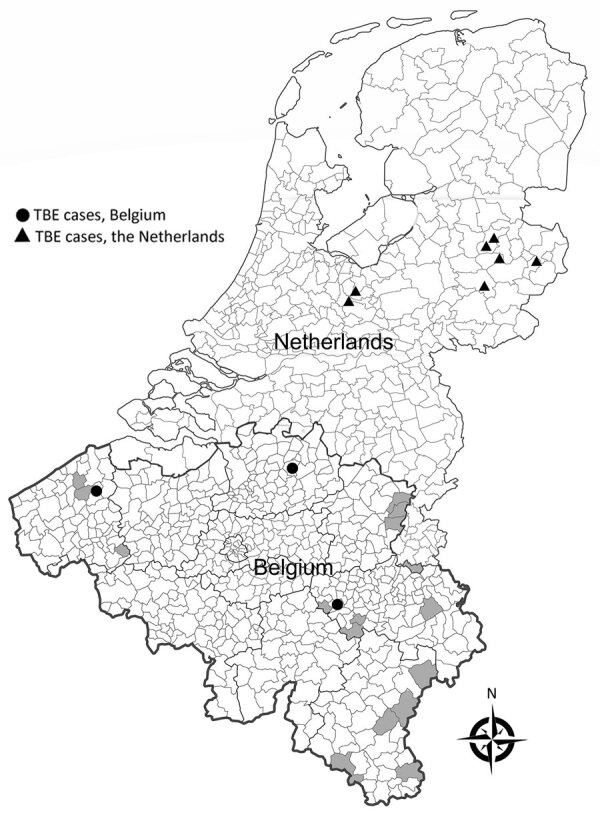
Geographic distribution of autochthonous human cases of tick-borne encephalitis, Belgium and the Netherlands (adapted from National Institute of Public Health and Environment [*10*]). Grey shading indicates communities in Belgium in which antibodies against tick-borne encephalitis virus have been detected in animals (adapted from S. Roelandt [*2*]).

TBE occurs after an incubation period of a median of 8 (range 4–28) days after a bite from an infected tick (*1*). Serologic diagnosis of TBE is hampered by a degree of cross reactivity with the antibodies against other flaviviruses in nearly all assays (*5*). The flavivirus IFA used by the NRC can determine the predominant flavivirus antibody response because it combines 8 different flavivirus substrates on different biochips. Unlike IgG, IgM responses generally are type-specific; therefore, IFA IgM is a useful tool for identification of infections during the acute phase of disease (*1*).

The incidence of TBE in Europe has increased in recent years, and the infection emerged in the Netherlands in 2016 and the United Kingdom in 2019 (*10*,*11*). The occurrence of autochthonous cases in the Netherlands in 2016, not far from the border with Belgium, led to a 26% increase in TBE serology inquiries at the NRC in 2017 compared with those for 2016 and a 143% increase in 2018 compared with those for 2017 (M. Van Esbroeck, unpub. data). In Belgium, the virus has been shown to circulate in animals, but human infections have been limited to a few imported cases until now (*2*,*12*). In 2018, two human cases of autochthonous TBE were suspected but not confirmed because both patients also spent time abroad during the incubation period (*3*,*13*,*14*). In a study on the prevalence of pathogens in ticks collected from humans in Belgium, none of the examined ticks were infected with TBEV (*14*,*15*). Studies to determine the geographic spread and genetic diversity of TBEV in ticks were put on hold in 2020 due to the COVID-19 pandemic. During the 2021 tick season, ticks will be collected by flagging in areas where TBEV exposure most likely occurred for the 3 described patients.

Confirmed TBE cases involving the central nervous system are reported to the European Surveillance System (*3*). Because approximately two thirds of human TBEV infections are asymptomatic, TBE probably is underdiagnosed in Europe (*15*).

Vaccination against TBEV is not recommended for the general population (*3*). However, persons living in Belgium should be aware of the risk for exposure to ticks and protect themselves against tick bites when engaging in outdoor activities. Clinicians also should include TBE in the differential diagnosis in patients with etiologically unexplained neurologic manifestations, even without a recent travel history.
